# Human iPSC derived organoid models to study tau pathology

**DOI:** 10.1002/alz.087353

**Published:** 2025-01-09

**Authors:** Taylor Bertucci, Kathryn Bowles, Steven Lotz, Le Qi, Katherine Stevens, Susan K Goderie, Susan Borden, Laura M Oja, Keith Lane, Ryan Lotz, Hailey Lotz, Rebecca Chowdhury, Shona Joy, Brigitte Arduini, David Butler, Michael Miller, Heide Baron, Carl Alexander Sandhof, M. Catarina Silva, Stephen Haggarty, Daniel H. Geschwind, Alison M. Goate, Sally Temple

**Affiliations:** ^1^ Neural Stem Cell Institute, Rensselaer, NY USA; ^2^ University of Edinburgh, Edinburgh, Scotland United Kingdom; ^3^ University of California, Los Angeles School of Medicine, Los Angeles, CA USA; ^4^ Icahn School of Medicine at Mount Sinai, New York, NY USA; ^5^ Massachusetts General Hospital, Boston, MA USA; ^6^ University of California, Los Angeles, Los Angeles, CA USA

## Abstract

**Background:**

Human pluripotent stem cell (hPSC)‐derived brain organoids patterned towards the cerebral cortex are valuable models of interactions occurring *in vivo* in cortical tissue. We and others have used these cortical organoids to model dominantly inherited FTD‐tau. While these studies have provided essential insights, cortical organoid models have yet to reach their full potential. Studies are hindered by well‐recognized hurdles: low production efficiency and high variability between individual organoids, across lines, and experiments. A protocol that consistently generates well‐patterned cerebral cortical organoids is needed.

**Methods:**

Two key stages of the protocol were optimized: the initial growth of hPSCs and the first 6 days of differentiation. Analysis of hPSC pluripotency and organoid quality control (QC) measures at 20 day and 2 months was assessed by bulk RNAseq, qPCR and IHC for ± QC and single‐cell RNA sequencing (scRNAseq). The optimized protocol was tested across 63 lines to examine robustness.

**Results:**

We established a 96 Slit‐well plate method for efficient (approaching 100%), scalable, reproducible cortical organoid production. When hPSCs were cultured with controlled‐release FGF2 (FGF2Discs) and an SB431542 concentration appropriate for their *TGFBR1*/*ALK5* expression level, organoid cortical patterning and reproducibility were significantly improved. Well‐patterned organoids included 16 neural subtypes identified by scRNA‐seq, abundant rosettes, and robust BCL11B+/TBR1+ cortical neurons at 2 months. In contrast, poorly patterned organoids contained mesendoderm‐related cells, identifiable by negative QC marker *COL1A2* and/or few cortical neurons. Using this improved protocol, we demonstrate increased sensitivity to study the impact of *MAPT* mutations associated with FTD, and across different *MAPT*‐mutants, neurons exhibited common early changes in key metabolic pathways, including upregulation of oxidative phosphorylation and dampening of ceramide synthesis and sphingolipid signaling.

**Conclusions:**

Variability between different hPSC lines in the expression of key ligands and receptors contributes to the variability of success of cortical organoid production. Hence, small adjustments in the culture of hPSCs and the concentration of patterning molecules during the first 6 days of differentiation are essential to enable efficient and reproducible organoid production. This allows rigorous comparisons of disease phenotypes using many different lines from different donors and across heritable and sporadic diseases affecting the cerebral cortex.

